# Estimating the Biodegradability of Treated Sewage Samples Using Synchronous Fluorescence Spectra

**DOI:** 10.3390/s110807382

**Published:** 2011-07-25

**Authors:** Tien M. Lai, Jae-Ki Shin, Jin Hur

**Affiliations:** 1 Department of Environment and Energy, Sejong University, 98 Gunja-dong, Gwangjin-gu, Seoul 143-747, Korea; E-Mail: minhtien1383@yahoo.com; 2 Korea Institute of Water and Environment, Korea Water Resources Corporation, Daejeon 306-711, Korea; E-Mail: jaekishin@kowaco.or.kr

**Keywords:** biodegradability, synchronous fluorescence spectrum, derivative spectroscopy, multiple regression analysis, wastewater

## Abstract

Synchronous fluorescence spectra (SFS) and the first derivative spectra of the influent *versus* the effluent wastewater samples were compared and the use of fluorescence indices is suggested as a means to estimate the biodegradability of the effluent wastewater. Three distinct peaks were identified from the SFS of the effluent wastewater samples. Protein-like fluorescence (PLF) was reduced, whereas fulvic and/or humic-like fluorescence (HLF) were enhanced, suggesting that the two fluorescence characteristics may represent biodegradable and refractory components, respectively. Five fluorescence indices were selected for the biodegradability estimation based on the spectral features changing from the influent to the effluent. Among the selected indices, the relative distribution of PLF to the total fluorescence area of SFS (Index II) exhibited the highest correlation coefficient with total organic carbon (TOC)-based biodegradability, which was even higher than those obtained with the traditional oxygen demand-based parameters. A multiple regression analysis using Index II and the area ratio of PLF to HLF (Index III) demonstrated the enhancement of the correlations from 0.558 to 0.711 for TOC-based biodegradability. The multiple regression equation finally obtained was 0.148 × Index II − 4.964 × Index III − 0.001 and 0.046 × Index II − 1.128 × Index III + 0.026. The fluorescence indices proposed here are expected to be utilized for successful development of real-time monitoring using a simple fluorescence sensing device for the biodegradability of treated sewage.

## 1. Introduction

The rapid and continuous monitoring of environmental samples is important for understanding their fate and the detrimental effects when they are exposed to different environments [1–3] Biodegradability of wastewater is a portion of the organic matter in the sample that can be easily removed by microorganisms [4]. Monitoring the biodegradability of effluent wastewater may provide a key element for pre-evaluation of the efficacy of further treatment processes as well as for assessing the degree of the potential pollution when the effluent is released to nearby watersheds. Biodegradable portion in wastewater is responsible for fast bacterial growth, which may lead to the deterioration of water quality in natural waters [5,6].

A number of available methods have been established for estimating biodegradability of wastewater samples. Among those, a ratio of biochemical oxygen demand (BOD) to chemical oxygen demand (COD) is one of the well-adopted surrogates for estimating the biodegradability [7]. However, the BOD test requires five days of incubation to obtain the results and it is often inaccurate at low concentrations [8]. Another problem may arise from the potential presence of heavy metals and toxic substances in wastewater because they may inhibit the biological oxidation of organic constitutes by bacteria, resulting in significant errors in the BOD measurements [9,10]. Meanwhile, the procedure of COD test is followed by the production of undesirable chemical wastes [11]. In this regard, the BOD/COD ratio is not appropriate for *in situ* monitoring of numerous samples, where detecting a quick response to the changes of the values and cost saving manner are essential [12,13].

In comparison, measuring dissolved organic carbon (DOC) concentrations after microbial degradation of soluble wastewater in batch culture or bioreactors has been considered as more precise method for determining the biodegradability. Biodegradable DOC (BDOC) value is typically determined by a difference between the initial and the final DOC concentrations after a certain period of incubation time. For example, Servais *et al.* [14] proposed a simple procedure to estimate BDOC and biodegradable particulate organic carbon in raw and treated wastewater based on 45-day batch incubation. More recently, Khan *et al.* [15] proposed an innovative procedure to reduce the colonization time for BDOC determination using a bioreactor consisting of two stages of immobilized microbial cells. However, the long time required for the determination and/or for colonization to stabilize, which can range from days to weeks still remains a drawback for application of those techniques as monitoring tools [16,17].

To overcome these disadvantages, alternative techniques based on optical sensors such as UV absorbance and fluorescence spectroscopy have emerged for rapid monitoring of wastewater BDOC [8]. For example, UV absorbance at a single wavelength between 254 nm and 280 nm has shown a strong linear correlation with DOC concentrations in water [13,18]. In wastewater, however, some parameters such as turbidity, iron and nitrate may absorb the light and thus they might interfere the determination of biodegradability [19,20]. In this sense, fluorescence spectroscopy may be superior to UV absorbance for monitoring wastewater organic matter due to its higher sensitivity, selectivity, and the precision. Recently, this technique has been used as an *in situ* monitoring tool for water and wastewater quality [21,22].

In relation to biodegradability of wastewater, a notable advantage of fluorescence spectroscopy is its ability to identify different organic matter components in wastewater samples. For example, Ahmad and Reynolds [23] applied synchronous fluorescence spectra (SFS) to sewage samples collected from three different treatment plants, and they demonstrated that the main peak around at 280 nm and 380 nm corresponded to biodegradable aromatic hydrocarbons and the non-biodegradable fraction, respectively. Fluorescence excitation-emission matrix (EEM) is also capable of characterizing the specific fluorescent fractions in organic compounds in wastewater [24]. However, EEM requires a number of sequential fluorescence emission scans at consecutively increasing excitation wavelengths, which may hamper development of a rapid sensing technique and derivation of the spectral data.

Derivative spectroscopy is known as a useful analytical technique for extracting qualitative and quantitative information from spectra consisting of unresolved and/or overlapping bands, which can be achieved by using the first or higher derivatives of the normal spectrum with respect to wavelengths [25,26]. Although using derivative spectroscopy may result in the decrease of signal-to-noise-ratios, it enables one to better detect a sharp band in a broad background, or a narrow shoulder on a broad main band [27]. In this study, SFS of wastewater from six different wastewater treatment plants (WWTPs) were analyzed to suggest the prediction indices for the biodegradability of treated sewage. The objectives of this study were: (1) to examine the characteristics of the SFS and the derivative spectra for a number of the wastewater samples; and (2) to suggest the optimum fluorescence indices for the prediction of biodegradability of treated sewage samples.

## 2. Experimental Section

### 2.1. Sample Collection and Preservation

Wastewater samples were collected in 2 L sterile polyethylene bottles, which were pre-cleaned in distilled water, from six different wastewater treatment plants that discharge into the Han River (Korea). The influent and the effluent sewages were sampled in April, July, and October, 2009 before the grit chamber and after the biological treatment processes, respectively. The facilities investigated have a wide range of the treatment capacities from 100 to 150,000 m^3^ day^−1^ (Table 1). Samples were refrigerated immediately upon return from the field and analyzed in a laboratory within 24 h for their fluorescence measurements.

### 2.2. Analytical Methods

The collected samples were first filtered through a 0.1 mm mesh sieve to remove large sized suspended solids. The concentrations of BOD, carbonaceous BOD (CBOD), and COD were determined using a standard method [29]. The samples were then filtered using a pre-ashed Whatman GF/F filter (effective pore size ∼0.7 μm) to separate them into the dissolved and the particulate phases. Concentrations of DOC were determined directly on acidified, air-sparged samples using a Shimadzu V-CPH analyzer. Particulate organic carbon (POC) concentrations were determined with a CHN element analyzer (Flash EA1112). BDOC and biodegradable POC (BPOC) were measured according to the method suggested by Servais *et al.* [14]. Briefly, water samples were incubated in each incubation flask at 20 °C in aerobic condition and subsamples for DOC and POC measurements were collected after incubation of 28 days.

The relative precision of the DOC and POC analyses were <3% and <2%, respectively, based on repeated measurements. Total organic carbon (TOC) and biodegradable TOC (BTOC) concentrations of the samples were calculated by the summation of the DOC and the POC concentrations, and the summation of BDOC and BPOC concentrations, respectively.

Fluorescence measurements were conducted using a luminescence spectrometer (LS-50B, Perkin-Elmer) equipped with a 20 kW xenon arc lamp. Slit bandwidths were adjusted to 10 nm for both excitation and emission. In this study, SFS for excitation wavelengths ranging from 250 to 600 nm at 0.5-nm steps were used with a constant offset (Δλ = 30 nm). The scanning speed was 250 nm/min. Previous studies demonstrated that SFS with the selected offset successfully captured various fluorescence components of wastewater DOM [30–32]. To limit second-order Raleigh scattering, a 290-nm cutoff filter was used for all samples. The fluorescence response to a blank solution (Milli-Q water) was subtracted from the spectra of each sample to correct for Raman spectral overlap. Finally, fluorescence intensities of the samples were normalized to units of quinine sulfate equivalents (QSE) using the fluorescence of a diluted series of quinine sulfate dehydrate in 0.05 M sulfuric acid at an excitation/emission of 350/450 nm [33]. Quinine sulfate standardization was conducted for each set of samples to account for variations in the fluorescence signal caused by instrument drift. They were also acidified to pH 3.0 to avoid the potential interference of metals [24]. Relative precisions of <2% were routinely obtained by repeating the absorption and fluorescence measurements.

To obtain additional information from the spectroscopy, first derivative fluorescence spectra (*i.e.*, dA(λ)/dλ) were calculated based on the method of Hur *et al.* [27] using OriginPro7.5 (Origin Lab Corporation). Briefly, the process involved a stepwise interval smoothing of the original zero-order spectra using a selected order polynomial function followed by differentiation of that function to obtain the derivative spectra. For the present study, we selected a constrained second-order polynomial function for data smoothing using a 25-data point interval to obtain the first derivative [27].

## 3. Results and Discussion

### 3.1. Synchronous Fluorescence Spectra Characteristics

**Figure 1.**
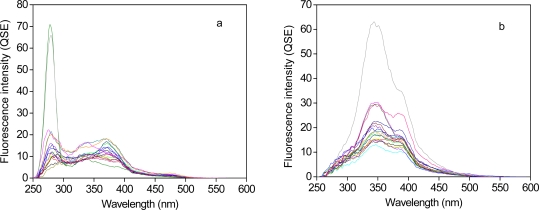
Synchronous fluorescence spectra of the influent (**a**) and the effluent (**b**) from WWTPs.

SFS of the influent and the effluent samples are shown in Figure 1. Three distinct fluorescence peaks were identified from the two types of the samples. The first peak, at the wavelengths between 250 nm and 300 nm, centered at ∼280 nm, was only observed for the influent samples. This peak, typically called protein-like fluorescence (PLF) peak, is related to the presence of aromatic amino acids and proteins such as tryptophan and tyrosine, and it is known to be associated with bacterial activity [31,34]. The second peak was detected at the wavelengths between 300 nm and 365 nm with the peak shifted toward a longer wavelength for the effluent *versus* the influent. This peak, denoted as fulvic-like fluorescence (FLF) peak, typically represents the characteristic fulvic acids [10,31,32] because it is commonly observed for surface water enriched with aquatic fulvic acids. The characteristics of the red-shifted FLF peak at the effluent has been utilized for tracking the source of effluent organic matter in the watersheds downstream of WWTP [35]. The last peaks are located at the wavelengths longer than 365 nm and those of the effluent were red-shifted. The presence of the peak may be attributed to more condensed humic-like fluorophores (HLF) contained in the samples [32,36]. The observation of the red-shifted peaks for the effluent suggests that structural changes in sewage may occur during the biological processes of the WWTP.

For the present study, the wavelength-dependent SFS was divided it into three main fluorescence regions including PLF, FLF, and HLF regions, each of which corresponds to the integrated areas of the fluorescence intensities at the wavelengths of 250–300 nm, 300–365 nm, and 365–600 nm, respectively.

Comparison of the SFS for the influent *versus* the effluent revealed substantial reduction of PLF region, enhancement of FLF region, and relatively small changes in HLF region after the wastewater treatment processes. These results are consistent with other previous reports on sewage DOM fluorescence [37–39]. For example, Janhom *et al.* [37] demonstrated that PLF characteristics of brewery wastewaters were preferentially removed by a biological treatment process consisting of upflow anaerobic sludge blanket and activated sludge. The enhancement of FLF was attributed therein to microbial production of fulvic-like components during the biological processes. Park *et al.* [38] confirmed using size exclusion chromatography that the enlargement of the FLF characteristics in sewage after a biological treatment process arise from the enrichment of more aromatic moieties. Recently, Guo *et al.* [39] have applied fluorescence EEM and parallel factor analysis for characterizing urban wastewater treated by the second aeration process, and they demonstrated a slight decrease in the PLF and the short wavelength-excited humic-like components (*i.e*., red-shifted).

### 3.2. First Derivative Synchronous Fluorescence Spectra

**Figure 2.**
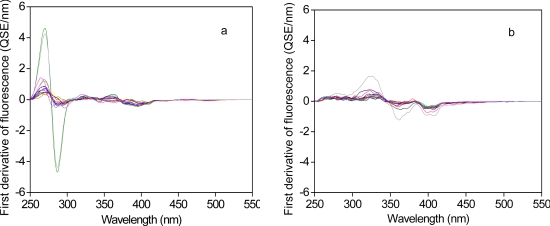
First derivative synchronous fluorescence spectra of the influent (**a**) and the effluent (**b**) from WWTPs.

Derivative spectroscopy techniques have been used to provide useful information on the changes in the structures and the composition of biological samples [40]. In this study, the first derivative synchronous fluorescence spectra of the influent and the effluent samples were obtained from the zero-order spectra following an established method by Hur *et al.* [27] (Figure 2). For the influent samples, one concave up and one concave down peak appeared consistently at the wavelengths of 270 nm and 290 nm, respectively. The relative differences between the two peak intensities are related to the sharpness of the PLF peak of the zero-order spectra with the higher values corresponding to the sharper PLF peaks. At the wavelengths longer than 300 nm, several small concave up and down peaks were found. Again, the relative differences between the maximum and the minimum values of the peaks in the wavelength range may represent the shape patterns of the FLF and the HLF peaks in the original spectra.

In contrast to the influent, for the effluent no obvious peaks were exhibited within the PLF region. One concave up and two concave down peaks were observed at the wavelengths between 300 nm and 350 nm, between 350 nm and 380 nm, and at around 400 nm, respectively. The first two peaks are associated with the enhanced FLF peak for the original spectra of the effluent samples. The last peak may be related to the presence of the shoulder at the wavelength of ∼400 nm for the original spectra, which was observed exclusively for the effluent samples.

Similar to the original spectra, comparison of the first derivative spectra for the influent *versus* the effluent revealed the spectral changes in the sewage organic matter by biological treatment processes. The spectral features in the PLF region became highly weakened whereas those of the FLF and HLF regions were relatively enhanced after biological treatment processes.

### 3.3. Selection of Spectral Features for the Prediction of Biodegradability in Sewage

Because the wastewater treatment plants investigated here are all based on biological treatment processes, the decreased and the enhanced spectral features from the influent to the effluent samples are possibly associated with biodegradable and refractory organic components of the treated sewage samples, respectively. In this context, PLF and HLF regions from the original SFS and the first derivative spectra may correspond to the biodegradable and the refractory types of the changed features, respectively. Therefore, the integrated area of the PLF region and its relative distribution to the total fluorescence area (*i.e*., %PLF) were first selected from the original spectra as Index I and Index II, respectively, for estimating biodegradability of sewage samples. In addition, an integrated area ratio of the PLF and the HLF regions for the original spectra was chosen for Index III. From the first derivative spectra, the integrated area of the PLF region (Index IV) and an integrated area ratio of the PLF and the HLF regions (Index V) were assigned to the prediction indices.

### 3.4. Comparison for the Prediction Capabilities of the Selected Estimation Indices

Biodegradability based on TOC measurements ranged from 0.06 to 0.44 with an averaged value of 0.24 (Table 2). The range falls within that of the traditional BOD/COD parameter, which corresponds to the values between 0.03 and 0.54. The ratios of CBOD to COD exhibited a shorter range from 0.03 to 0.27 compared to the BOD/COD ratios although the two parameters are significantly correlated each other (r = 0.791, p < 0.001). The different ranges for the two parameters (*i.e*., BOD/COD and CBOD/COD) suggest that a significant amount of nitrogenous oxygen demand (NOD) may be involved in the BOD results of the treated sewage, leading to the overestimation of the biodegradability.

**Figure 3.**
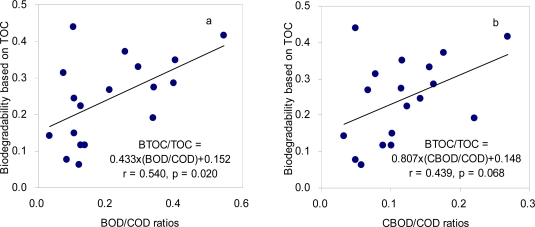
Correlations between TOC-based biodegradability and BOD/COD ratios (**a**) and between TOC-based biodegradability and CBOD/COD ratios (**b**).

The ratios of CBOD/COD, despite the general consensus that CBOD can more accurately describe biodegradable organic matters, did not show a better correlation with the TOC-based biodegradability compared to the BOD/COD ratios (Figure 3). This result indicates that both oxygen demand-based measurements for biodegradability still have limitations for accurately describing the biodegradable portion of the total organic matter in the treated sewage. The incubation time of five days appears to be insufficient for the complete biodegradation and the degradation rates may be affected by the composition of the treated sewage. For example, Gourlay *et al.* [41] demonstrated that aromatic carbon content of artificial wastewater DOM was associated with a lower degree of the biodegradability. In addition, chemical oxidation efficiency of COD test may be affected by the structural properties of organic matter samples and other environmental factors. Kylefors *et al.* [42] have shown that the interaction of other inorganic and organic substances with leachate DOM may result in an increase of the COD value.

**Figure 4.**
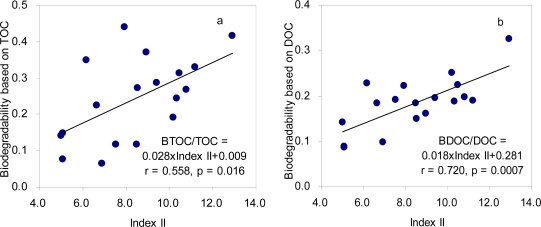
Correlation between Index II and TOC-based biodegradability (**a**) and between Index II and DOC-based biodegradability (**b**).

Correlation coefficients between the selected fluorescence indices and BTOC/TOC or BDOC/DOC ratios are listed in Table 3. The highest correlations for the biodegradability among the selected indices were obtained for Index II. The relative distributions of the PLF region for most samples appear to consistently decrease after biodegradation, probably due to the decrease of the PLF region and/or the enhancement of the FLF and the HLF regions. The correlation coefficients were 0.558 and 0.720 for TOC- and DOC-based biodegradability, respectively, which were even higher than those obtained with the BOD/COD ratios (Figure 4). It is notable that a significant regression equation was obtained despite the relatively short range of the DOC-based biodegradability. Our results revealed that the selected fluorescence feature could be successfully utilized as an estimation index for biodegradability of treated sewage, particularly for the dissolved phase. The higher estimation capability for the DOC-based biodegradability may be attributed to the measurement of the filtered samples for the fluorescence indices.

### 3.5. Improvement of the Biodegradability Estimation Using Multi-Regression Analysis

**Figure 5.**
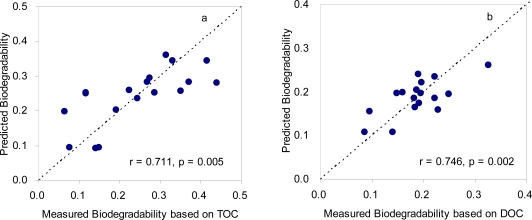
Correlation between measured TOC-based biodegradability (**a**) and DOC-based biodegradability (**b**) and the predicted values by using a multiple regression method based on Index II and Index III.

In order to improve the estimation capability of the fluorescence indices, multiple regression analyses were employed using the two fluorescence indices that exhibited the most significant correlations with the biodegradability among the tested indices (*i.e*., Index II and Index III). As a result, a clear enhancement in the correlations was observed compared to those obtained using a single index. The correlation coefficients increased from 0.558 to 0.711 and from 0.720 to 0.746 for TOC- and DOC-based biodegradability, respectively (Figure 5). These results revealed that the multiple regression method might be more advantageous for estimation of biodegradability in the effluents. The improvement was more pronounced for TOC-based biodegradability, indicating that the fluorescence feature associated with Index III (*i.e*., HLF) may partially account for the presence of particulate organic matter in the treated sewage despite the reflection of the dissolved phase. In related studies, Lee and Ahn [11] have demonstrated that fluorescence intensities at long wavelength may be associated with the concentrations of suspended solids in sewage. Wang and Zhang [43] implied in a study of fluorescence characterization of soluble microbial products that the PLF and HLF characteristics of effluent DOM might be enhanced due to high presence of suspended sludge.

A multiple regression based on Index II and Index III was established to improve the biodegradability estimation capability. The final multiple regression equations were 0.148 × Index II − 4.964 × Index III − 0.001 and 0.046 × Index II − 1.128 × Index III + 0.026 for TOC- and DOC-based biodegradability of treated sewage samples, respectively. It is notable that the estimation capability only based on fluorescence features was much higher than those with the traditional parameters, the BOD/COD or the CBOD/COD ratios. Although the fluorescence data obtained here are based on a standard laboratory instrument, it is expected that our proposed procedure here will be applied for developing *in situ* real-time monitoring of wastewater biodegradability. It is known that fluorescence sensing devices are easy to make in different sizes for flow injection systems. For successful development of fluorescence sensing device for biodegradability, fluorescence signals accounting for the presence of particulate matters in treated sewage need to be explored in the future work.

## 4. Conclusions

Three distinct peaks were identified from the SFS of wastewater samples for the influent and the effluent. PLF was consistently reduced whereas both FLF and HLF were enhanced during the biological treatment processes employed in the studied WWTPs. From the spectral changes from the influent and the effluent, PLF-related and FLF-or HLF-associated features were incorporated into the estimation indices for the biodegradability of the treated sewage samples. Among the selected indices, Index II exhibited the highest correlation coefficient with TOC-based biodegradability, which was even higher than those obtained with the BOD/COD or CBOD/COD ratios. A multiple regression analysis using Index II and Index III demonstrated the enhancement of the correlations from 0.558 to 0.711 and from 0.720 to 0.746 for TOC- and DOC-based biodegradability, respectively. The multiple regression equations finally suggested were 0.148 × Index II − 4.964 × Index III − 0.001 and 0.046 × Index II − 1.128 × Index III + 0.026 for TOC- and DOC-based biodegradability of treated sewage samples, respectively. The fluorescence indices and the associated procedures proposed here are expected to be utilized for the successful development of real-time monitoring using simple fluorescence sensing devices.

## Figures and Tables

**Table 1.** Summary of wastewater treatment plants.

**WWTP name**	**Type of wastewater**	**Treatment capacity (m^3^ day^−1^)**	**Treatment processes**
**JC**	Domestic sewage	70,000	Activated sludge process
**WJ**	Domestic sewage	13,000	Activated sludge with extended aeration process
**CC**	Domestic sewage	150,000	Activated sludge 4 stage BNR (Biological Nutrient Removal)
**CJ**	Domestic sewage with some landfill leachate	75,000	B3 process ^a^
**YI**	Domestic sewage with some livestock waste	48,000	B3 process ^a^
**HS**	Livestock waste	100	Activated sludge with extended aeration process

a The details of the process are described in Kim *et al.* [28].

**Table 2.** Statistical summary for the biodegradability and the selected fluorescence indices for the effluent samples in this study (*n* = 18).

	
	**Mean**	**SD**	**Range**
Ratio of biodegradability			
**BOD/COD**	0.21	(0.14) *	0.03–0.54
**CBOD/COD**	0.12	(0.06)	0.03–0.27
**BTOC/TOC**	0.24	(0.12)	0.06–0.44
**BDOC/DOC**	0.18	(0.06)	0.09–0.33
Fluorescence indices			
**Index I**	344.51	(90.62)	225.89–520.11
**Index II**	8.42	(2.32)	5.01–12.91
**Index III**	0.20	(0.06)	0.13–0.31
**Index IV**	16.87	(4.41)	9.67–30.41
**Index V**	0.48	(0.11)	0.30–0.64

* The numbers in the parentheses represent standard deviation.

**Table 3.** The correlation coefficients between the selected fluorescence indices and various types of biodegradability parameters for the effluent.

	
	**Index I**	**Index II**	**Index III**	**Index IV**	**Index V**
**BOD/COD**	0.297 (0.231) *	0.537 (0.021)	0.515 (0.029)	0.027 (0.915)	0.507 (0.031)

**CBOD/COD**	0.299 (0.227)	0.643 (0.004)	0.657 (0.003)	0.032 (0.898)	0.636 (0.005)

**BDOC/DOC**	0.442 (0.066)	0.720 (<0.001)	0.674 (0.002)	0.146 (0.564)	0.648 (0.004)

**BTOC/TOC**	0.364 (0.137)	0.558 (0.016)	0.470 (0.048)	−0.002 (0.994)	0.347 (0.158)

* The numbers in the parentheses are p-values.

## References

[1] Kim KH, Mishra VK, Hong SM (2006). The rapid and continuous analysis of mercury behavior in ambient air. Atmos. Environ.

[2] Kim KH (2006). Emissions of reduced sulfur compounds (RSC) as a landfill gas (LFG): A comparative study of young and old landfill facilities. Atmos. Environ.

[3] Nguyen HT, Kim KH, Kim MY, Hong S, Youn YH, Shon ZH, Lee JS (2007). Monitoring of atmospheric mercury at a Global Atmospheric Watch (GAW) site on An-Myun Island, Korea. Water Air Soil Pollut.

[4] Tusseau-Vuillemin MH, Dispan J, Mouchel JM, Servais P (2003). Biodegradable fraction of organic carbon estimated under oxic and anoxic conditions. Water Res.

[5] Servais P, Anzil A, Ventresque C (1989). Simple method for determination of biodegradable dissolved organic carbon in water. Appl. Environ. Microbiol.

[6] Escobar IC, Randall AA, Taylor JS (2001). Bacterial growth in distribution systems: Effect of assimilable organic carbon and biodegradable dissolved organic carbon. Environ. Sci. Technol.

[7] Amat AM, Arques A, García-Ripoll A, Santos-Juanes L, Vicente R, Oller I, Maldonado MI, Malato S (2009). A reliable monitoring of the biocompatibility of an effluent along an oxidative pre-treatment by sequential bioassays and chemical analyses. Water Res.

[8] Bourgeois W, Burgess J, Stuetz RM (2001). On-line monitoring of wastewater quality: A review. J. Chem. Technol. Biotechnol.

[9] Qian Z, Tan TC (1999). BOD measurement in the presence of heavy metal ions using a thermally-killed-Bacillus subtilis biosensor. Water Res.

[10] Hur J, Kong DS (2008). Use of synchronous fluorescence spectra to estimate biochemical oxygen demand (BOD) of urban rivers affected by treated sewage. Environ. Technol.

[11] Lee S, Ahn KH (2004). Monitoring of COD as an organic indicator in waste water and treated effluent by fluorescence excitation-emission (FEEM) matrix characterization. Water Sci. Technol.

[12] Liu J, Olsson G, Mattiasson B (2004). Short-term BOD (BODst) as a parameter for on-line monitoring of biological treatment process: Part I. A novel design of BOD biosensor for easy renewal of bio-receptor. Biosens. Bioelectron.

[13] Nataraja M, Qin Y, Seagren EA (2006). Ultraviolet spectrophotometry as an index parameter for estimating the biochemical oxygen demand of domestic wastewater. Environ. Technol.

[14] Servais P, Barillier A, Garnier J (1995). Determination of the biodegradable fraction of dissolved and particulate organic carbon in waters. Ann. Limnol. Int. J. Lim.

[15] Khan E, Babcock RW, Jongskul S, Devadason FA, Tuprakay S (2003). Determination of biodegradable dissolved organic carbon using entrapped mixed microbial cells. Water Res.

[16] Kaplan LA, Newbold JD (1995). Measurement of streamwater biodegradable dissolved organic carbon with a plug-flow bioreactor. Water Res.

[17] Søndergaard M, Worm J (2001). Measurement of biodegradable dissolved organic carbon (BDOC) in lake water with a bioreactor. Water Res.

[18] Brookman SKE (1997). Estimation of biochemical oxygen demand in slurry and effluents using ultra-violet spectrophotometry. Water Res.

[19] Wang GS, Hsieh ST (2001). Monitoring natural organic matter in water with scanning spectrophotometer. Environ. Int.

[20] Weishaar JL, Aiken GR, Bergamaschi BA, Fram MS, Fujii R, Mopper K (2003). Evaluation of specific ultraviolet absorbance as an indicator of the chemical composition and reactivity of dissolved organic carbon. Environ. Sci. Technol.

[21] Hudson N, Baker A, Reynolds D (2007). Fluorescence analysis of dissolved organic matter in natural, waste and polluted waters—A review. River Res. Appl.

[22] Henderson RK, Baker A, Murphy KR, Hambly A, Stuetz RM, Khan SJ (2009). Fluorescence as a potential monitoring tool for recycled water systems: A review. Water Res.

[23] Ahmad SR, Reynolds DM (1995). Synchronous fluorescence spectroscopy of wastewater and some potential constituents. Water Res.

[24] Westerhoff P, Chen W, Esparza M (2001). Fluorescence analysis of a standard fulvic acid and tertiary treated wastewater. J. Environ. Qual.

[25] Bosch Ojeda C, Sanchez Rojas F (2004). Recent developments in derivative ultraviolet/visible absorption spectrophotometry. Anal. Chim. Acta.

[26] Sánchez Rojas F, Bosch Ojeda C (2009). Recent development in derivative ultraviolet/visible absorption spectrophotometry: 2004–2008: A review. Anal. Chim. Acta.

[27] Hur J, Williams MA, Schlautman MA (2006). Evaluating spectroscopic and chromatographic techniques to resolve dissolved organic matter via end member mixing analysis. Chemosphere.

[28] Kim JK, Park KJ, Cho KS, Nam SW, Park TJ, Bajpai R (2005). Aerobic nitrification-denitrification by heterotrophic Bacillus strains. Bioresour. Technol.

[29] American Public Health Association, American Water Works Association, Water Environmental Federation (2005). Standard Methods for the Examination of Water & Wastewater.

[30] Hur J, Lee BM, Lee TH, Park DH (2010). Estimation of biological oxygen demand and chemical oxygen demand for combined sewer systems using synchronous fluorescence spectra. Sensors.

[31] Jaffé R, Boyer JN, Lu X, Maie N, Yang C, Scully NM, Mock S (2004). Source characterization of dissolved organic matter in a subtropical mangrove-dominated estuary by fluorescence analysis. Mar. Chem.

[32] Hur J, Hwang SJ, Shin JK (2008). Using synchronous fluorescence technique as a water quality monitoring tool for an urban river. Water Air Soil Pollut.

[33] Chen Z, Hu C, Conmy RN, Muller-Karger F, Swarzenski P (2007). Colored dissolved organic matter in Tampa Bay, Florida. Mar. Chem.

[34] Chen W, Westerhoff P, Leenheer JA, Booksh K (2003). Fluorescence excitation-emission matrix regional integration to quantify spectra for dissolved organic matter. Environ. Sci. Technol.

[35] Baker A (2001). Fluorescence excitation-emission matrix characterization of some sewage-impacted rivers. Environ. Sci. Technol.

[36] Chen J, LeBoeuf EJ, Dai S, Gu B (2003). Fluorescence spectroscopic studies of natural organic matter fractions. Chemosphere.

[37] Janhom T, Wattanachira S, Pavasant P (2009). Characterization of brewery wastewater with spectrofluorometry analysis. J. Environ. Manage.

[38] Park MH, Lee TH, Lee BM, Hur J, Park DH (2009). Spectroscopic and chromatographic characterization of wastewater organic matter from a biological treatment plant. Sensors.

[39] Guo W, Xu J, Wang J, Wen Y, Zhuo J, Yan Y (2010). Characterization of dissolved organic matter in urban sewage using excitation emission matrix fluorescence spectroscopy and parallel factor analysis. J. Environ. Sci.

[40] Mozo-Villarías A (2002). Second derivative fluorescence spectroscopy of tryptophan in proteins. J. Biochem. Bioph. Meth.

[41] Gourlay C, Tusseau-Vuillemin MH, Mouchel JM, Garric J (2005). The ability of dissolved organic matter (DOM) to influence benzo[a]pyrene bioavailability increases with DOM biodegradation. Ecotox. Environ. Safe.

[42] Kylefors K, Ecke H, Lagerkvist A (2003). Accuracy of COD test for landfill leachates. Water Air Soil Pollut.

[43] Wang ZP, Zhang T (2010). Characterization of soluble microbial products (SMP) under stressful conditions. Water. Res.

